# A randomized controlled trial of cognitive control training (CCT) as an add-on treatment for late-life depression: a study protocol

**DOI:** 10.1186/s12888-021-03597-1

**Published:** 2021-11-27

**Authors:** Bart Meuleman, Janna N. Vrijsen, Marie-Anne Vanderhasselt, Ernst H. W. Koster, Peter Oostelbos, Paul Naarding, Linda Bolier, Indira Tendolkar, Filip Smit, Jan Spijker, Eni S. Becker

**Affiliations:** 1 Depression Expertise Centre, Pro Persona Mental Health Care, Nijmeegsebaan 61, 6525 DX Nijmegen, the Netherlands; 2 Behavioural Science Institute, Radboud University Nijmegen, Nijmegen, the Netherlands; 3 Donders Institute for Brain, Cognition and Behaviour, Department of Psychiatry, Radboud University Medical Center, Nijmegen, the Netherlands; 4 Department of Experimental Clinical and Health Psychology, Ghent University, Ghent, Belgium; 5 Dutch Depression Association, Amersfoort, The Netherlands; 6 De Hartenboom, Randwijk, the Netherlands; 7 GGNet Network for Mental Health Care, Zutphen, the Netherlands; 8 Trimbos Institute (Netherlands Institute of Mental Health and Addiction), Utrecht, the Netherlands; 9 Department of Epidemiology and Biostatistics, University Medical Centers Amsterdam, Location VUmc, Amsterdam, the Netherlands; 10 Department of Clinical Psychology, University Medical Centers Amsterdam, Location VUmc, Amsterdam, the Netherlands

**Keywords:** Cognitive control, Late-life depression, (cost-)effectiveness

## Abstract

**Background:**

Already a major health concern, late-life depression (LLD) is expected to form an increasing problem in the aging population. Moreover, despite current treatments, LLD is associated with a poor long-term prognosis and high rate of chronicity. Treatment provision and treatment accordingly warrant improvement, where add-on treatments might contribute to the efficacy of conventional therapies. Although it is known that impaired cognitive control contributes to LDD, it is not targeted sufficiently by current interventions. Research on cognitive control training (CCT) shows promising results on depressive symptoms, cognitive performance, and overall functioning. However, further research is needed to determine the long-term effects of CCT on LLD, its cost-effectiveness, and mechanisms of change.

**Methods:**

In the current multicenter randomized controlled trial (RCT) with a between-subjects design participants aged 60 years and over with a current LLD receiving treatment as usual (TAU) are randomized to add-on CCT or placebo training. Randomization is stratified by depression severity. Participants will receive eight online CCT or placebo sessions spread across four consecutive weeks. They will complete a post-training assessment after 1 month and three follow-up assessments scheduled three, six and 12 months after completing the training. We expect CCT and TAU to be more (cost-)effective in reducing depressive symptoms than placebo training and TAU. Additionally, we will be looking at secondary clinical, cognitive and global functioning outcomes and likely mechanisms of change (e.g., improved cognitive functioning, reduced rumination, and improved inhibition of negative stimuli).

**Discussion:**

The proposed RCT aims to contribute to the clinical and scientific knowledge on the long-term effects of CCT as an add-on treatment for LLD. Cost-effectiveness is particularly relevant considering the expected volume of the target demographic. The study will be a pragmatic trial with few inclusion restrictions, providing information on feasibility of web-based trainings in clinical settings. The outcomes are potentially generalizable to guidelines for treatment of LLD.

**Trial registration:**

This trial is registered in the Netherlands Trial Register (code: NL7639). Registered 3 april 2019.

## Background

Late-life depression (LLD), defined as major depressive disorder in individuals over 60 years of age, is a severe psychiatric illness, and has a prevalence ranging between 1 and 16% [20]. LLD has a poor long-term prognosis, and is associated with chronicity and high mortality [32, 34, 39]. Research shows that relapse, recurrence, and chronicity are higher in LLD than they are in younger populations [25], while in later life longer duration and chronicity are associated with further cognitive decline and somatic comorbidity such as cerebrovascular diseases and diabetes [2, 15, 47]. Due to aging of the global population, the burden associated with LLD is expected to increase further, which underscores the necessity of appropriate, evidence-based, and cost-effective treatments that are tailored to older populations [9].

Older adults with depression seem less responsive to standard treatment protocols than their younger counterparts. The Netherlands Study of Depression in Older Persons (NESDO) reported that only 18% of their participants diagnosed with major depressive disorder (MDD) had recovered after 2 years [15], despite having received psychopharmacological and psychotherapeutic treatment from primary or specialized mental health-care services. In comparison, approximately 80% of a younger adult population recovered in a two-year period [57]. Moreover, the efficacy of antidepressant medication is lower in older adults than it is in younger adults, which is possibly related to age-related cognitive impairments [4, 49]. For example, a meta-analysis of 74 studies showed that the number needed to treat for antidepressants was six for adults, eight for persons over 55 years and 21 for people over 65 years [59]. Furthermore, for older depressed patients access to therapy is limited [16] and treatment often does not take into account underlying age-specific contributing factors. Arguably, add-on treatments aimed at distinct mechanisms of LLD may improve outcomes in this population.

Impairment in a broad range of cognitive functions [51] is a common and disabling factor in LLD [42]. Apart from manifesting in performance tasks, cognitive deficits have also been observed in the brain. On a structural level, temporary changes in frontal brain regions are related to depressive symptomatology [1, 42] but particularly relevant are the changes discerned in the cognitive control network (CCN), which consists of the dorsolateral prefrontal cortex (DLPFC), the dorsal anterior cingulate cortex (dACC), and parietal regions [3]. The CCN plays a crucial role in emotion regulation and abnormalities in the associated regions can result in weakened cognitive control over negative affect [1, 3]. Cognitive control is implicated in a range of cognitive functions, such as working memory, attention span, and executive functions, loss of which can cause (increased) dysfunctional repetitive negative thinking, eliciting or aggravating depressive symptoms [18]. This specific vulnerability pathway has been supported by multiple MDD studies in adults, underscoring the importance of dedicated interventions targeting this crucial function [3, 8, 19]. Furthermore, in LLD the prevalence of impaired cognitive control functions is particularly high, which has also been associated with the reduced effectiveness of antidepressants [4, 42, 49]. An added treatment that specifically targets impaired cognitive control in older adults coping with depression may then be the preferred approach.

In recent years, researchers have started evaluating computerized cognitive control training (CCT) programs. These interventions generally consist of repetitive performance of cognitive tasks, aiming to (re)activate specific processes that potentially help restore the cognitive function addressed. In general, CCT is associated with small to moderate improvements of cognitive skills, depressive symptoms, and global functioning (see [35], and meta-analyses by Motter et al. [43]; Legemaat et al. [36]). These effects seem to complement the effects of treatment as usual (TAU). More specifically, CCT programs seem to be an effective and acceptable intervention for depressed older adults [43]. Additionally, online CCT is a low-cost, highly scalable treatment option. However, the number of studies examined was small and the authors did stress the need to further examine whether the cognitive gains spread to other neurocognitive processes, symptoms, and overall functioning. Relatedly, a systematic review of different types of CCT for healthy older participants shows that after training specific executive functions, small to moderate gains in cognitive performance are observed [45]. Interestingly, CCT appears especially effective when the applied tasks are continuously adapted to the participants’ performance level. Therefore, adding an adaptive form of CCT to current treatment of depressed older adults, might result in beneficial effects on cognitive functions, depressive symptoms and overall functioning. A randomized controlled trial (RCT) comparing such an adaptive CCT format to placebo add-on treatment in specialized health care would be the appropriate means to further test this.

The adaptive Paced Auditory Serial Addition Task (aPASAT) is a promising intervention aimed at enhancing cognitive control [24, 56]. In this task, individuals are instructed to sum up the last two numbers they heard in an auditorily presented continuous stream of numbers. It is adaptive in that its presentation speed is continuously matched to the participant’s performance, inducing greater emotional reactivity (e.g., frustration, negative thoughts, some negative affect), which might account for the benefits derived from such tailored tasks [11, 17]. Siegle et al. [56] concluded that the aPASAT activates the cognitive control network by showing that the dorsolateral prefrontal cortex is engaged during its performance. They also tested the task in participants with MDD, in whom the cognitive training reduced depressive symptoms and rumination more so than was the case in a control group receiving treatment as usual (TAU) [56]. Further studies continued to show beneficial effects of the aPASAT on depressive symptoms in various samples, where gains were possibly mediated by diminished rumination through improved cognitive functioning [12, 53, 60]. Interestingly, Brunoni et al. [12] observed, in a combined treatment group receiving CCT and transcranial direct-current stimulation, that sustained reduction in depressive symptoms was greatest in the older participants, even when controlling for changes in cognitive control throughout the training. This suggests that especially older depressed adults benefit from the aPASAT as an add-on to other treatment.

In summary, there is an urgent need for innovative, effective, and affordable treatments for LLD, which can be delivered alongside treatment as usual. The literature indicates that adaptive CCT approaches may be most effective in improving cognitive functioning and reducing rumination in older adults. The aPASAT is a relatively low-cost intervention that can be used as an add-on treatment to TAU, but large-scale clinical trials investigating its (cost-)effectiveness, working mechanisms, and feasibility in LLD are lacking. We aim to address these questions in the RCT described below.

### Aims and hypotheses

Our primary objective is to investigate whether the addition of a CCT program to ongoing LLD treatment (i.e., pharmacotherapy and/or psychotherapy) will help reduce depressive symptoms more so than a placebo training. Outcomes will be assessed 1 month post-training (T1) and during follow-up assessments at three (T2), six (T3) and 12 months (T4). We expect symptom reductions in the participants having received CCT to be greater than in the participants having completed the placebo training at all timepoints. Our second aim is to see whether CCT also exerts effects on other clinical measures including rumination (both trait and state), cognitive emotion regulation, working memory functioning (near and far transfer), inhibition, and quality of life. We expect that participants in the intervention group will show lower rumination, more adaptive cognitive emotion regulation, more improved working memory functioning, inhibition, and quality of life than the participants in the placebo group. Our third aim is to identify working mechanisms of CCT. We will examine whether improved working memory and reduced rumination mediate the effects of CCT on depressive symptoms, hypothesizing that improvements in depressive symptoms will follow improvement of working memory and reduction of rumination in the intervention group. Fourthly, we aim to study individual differences that could account for differences in the effect of CCT on symptom change, where we expect that baseline cognitive control, depression severity, and age will be moderating factors. Our fifth aim is to determine the cost-effectiveness of our add-on CCT, we will conduct a health-economic evaluation and budget-impact analysis, anticipating that CCT will reduce mental healthcare consumption during the follow-up period more so than placebo training. Finally, for our sixth aim, we will be assessing the acceptability and feasibility of the intervention by conducting qualitative interviews with subsamples of participants having received CCT, therapists involved in mental health care for older persons and other stakeholders to inform the further implementation of CCT as an add-on intervention next to existing treatments for LLD.

## Methods

### Design

Our study comprises a single blind RCT with a between-subjects design and two conditions. Participants are randomly allocated to the intervention condition (CCT) or the active control condition (placebo training). Qualitative interviews will be conducted in two subgroups of participants in the intervention arm.

### Participants

The study and control sample will both include adults aged 60 years and over with a current MDD diagnosis according to the DSM-IV (Diagnostic and Statistical Manual of Mental Disorders [5] receiving inpatient or outpatient treatment at three regional mental-health patient centers (Pro Persona, GGNet, and Senior Beter) or the Radboud University Medical Center, Nijmegen, the Netherlands, all specialized in the treatment of late-life depression. Depression diagnoses and exclusion criteria will be confirmed by trained clinicians using structured diagnostic interviews, either the Mini International Neuropsychiatric Interview (M.I.N.I.) [54] or the Structured Clinical Interview (SCID-I) [22]. Ongoing depression treatment can consist of a combination of pharmacotherapy, psychotherapy (e.g., cognitive-behavioral therapy or interpersonal psychotherapy) or psychosocial treatment, varying in frequency. Therapy can be adapted during the trial, with changes being documented by the researchers.

Exclusion criteria are:

- Any current (features of) psychotic disorder (according to the DSM-IV)
- A current (hypo)mania or a history of bipolar disorder (DSM-IV)
- A primary diagnosis of substance or alcohol dependence or abuse (DSM-IV)
- Acute suicidal risk, assessed by a trained clinician
- Insufficient command of the Dutch language hindering participation in the study intervention
- Sensorimotor disabilities that can interfere with task performance (computer-based CCT)
- Severe cognitive disabilities interfering with therapy / CCT participation as assessed by a clinician. In doubt, a Minimal Mental State Exam (MMSE) will be administered, where a score below 18 will serve as the threshold for exclusion [33]

### Procedure

#### Recruitment, baseline assessment and randomization

**Fig. 1:**
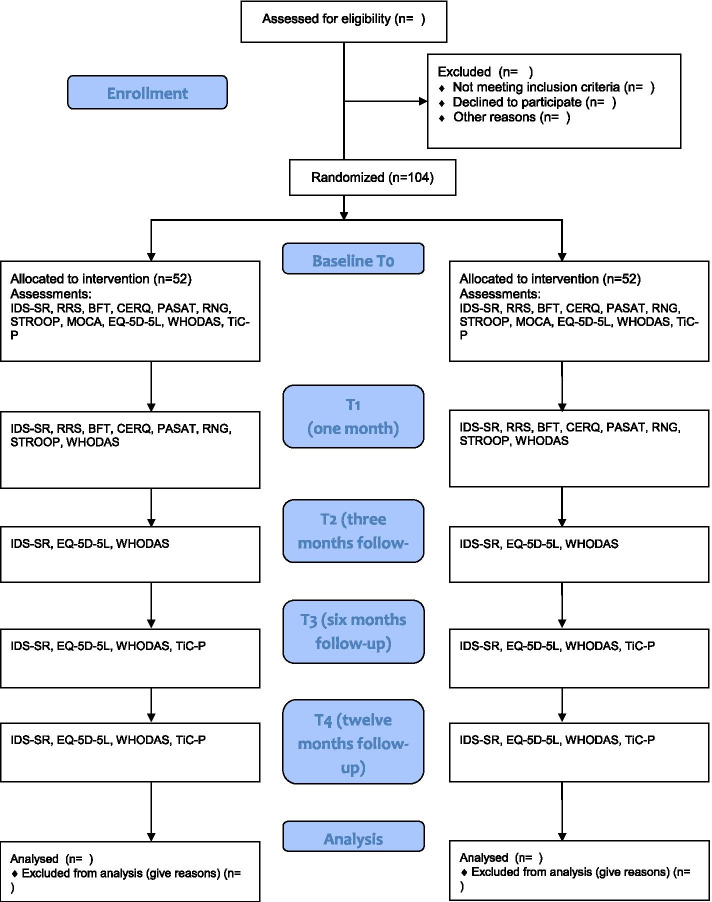
CONSORT flow chart

A depression experience expert (author PO) provided input on the intervention and application procedure before the start of the trial. The recruitment and assessment process is illustrated in a flowchart shown in Fig. 1. Clinicians will provide eligible candidates with basic information about the study on paper and/or through an informative video. Clinicians will only inform patients capable of consenting to participation. Interested candidates are referred to the researchers who will provide further information by phone. Eligible participants will be asked to provide their written and informed consent for participation at their first visit to the treatment site, where they will subsequently complete the baseline assessment (T0) gauging depressive symptoms, cognitive functioning, rumination, cognitive emotion regulation, quality of life, and health-care consumption, and health-related disability (economic evaluation). If inclusion criteria are satisfied, participants are randomly assigned to the intervention (CCT) or the active control condition (placebo training) by computer-generated allocation stratified for depression severity as assessed with the Inventory of Depressive Symptomatology Self Report: mild (IDS-SR < 38), moderate (IDS 39–48) and severe (IDS > 49) [52]. At the end of the baseline assessment, all participants are provided with a digital tablet to enable them to complete the eight training sessions in their own homes. Researchers will explain, demonstrate and practice the use of the tablet and schedule the training sessions together with the participant. During the one-month training period, participants will receive email notifications to remind them of the task, while researchers will regularly contact them by phone to monitor their progress, with the number and timepoint of the telephone contacts being documented for each participant.

Approximately 1 month after the training, a post-training evaluation (T1) will be conducted including all but three of the baseline measurements. For an overview of the measurements and their timepoints, we refer to Table 1. Participants are asked whether any changes have occurred in the LLD treatment during the training period. Participants that did not complete all training sessions will also be asked to complete all post- and follow-up assessments.

**Table 1:** Overview of measures and corresponding timepoints

Measure	Target concept	Baseline (T0)	Post-training (T1)	FU-1 (T2)	FU-2 (T3)	FU-3 (T4)
**IDS-SR**	Severity of depressive symptoms	●	●	●	●	●
**RRS**	Trait rumination	●	●			
**BFT**	State rumination	●	●			
**CERQ**	Cognitive emotion regulation strategies	●	●			
**PASAT**	Working memory capacity	●	●			
**RNG Task**	Working memory capacity	●	●			
**STROOP**	Inhibition of interfering stimuli	●	●			
**MOCA**	Cognitive dysfunction	●				
**EQ-5D-5l**	Health-related quality of life	●		●	●	●
**WHODAS 2.0**	Functioning and disability	●	●	●	●	●
**TiC-P**	Health-care costs (in the Netherlands) based on self-reported health consumption and productivity loss	●			●	●

*IDS-SR* Inventory of Depressive Symptomatology, *RRS* Ruminative Response Scale, *BFT* Breathing Focus Task, *CERQ* Cognitive Emotion Regulation Questionnaire, *PASAT* Paced Auditory Serial-Addition Task, *RNG task* Random Number Generation task, *Stroop task* (i.e. inhibition of interfering stimuli), *MOCA* Montreal Cognitive Assessment, *EQ-5D-5L* (i.e. quality of health), *WHODAS* World Health Organization Disability Assessment Schedule, *TiC-P* Trimbos Institute and iMTA Cost questionnaire for Psychiatry (i.e. economic costs)

Participants will again fill out the questionnaires 3, 6 and 12 months (T2, T3, and T4) post-training. Health-care utilization data will be collected with the TiC-P at T0, T3 and T4. The qualitative interviews for the flanking implementation study will be conducted between the post-training and 3-month follow-up. For a complete overview of the study design, we refer to Fig. 1.

Because of the COVID-19 pandemic, we created an online version of the original study procedure to enable us to safely test participants. The baseline and post-training assessment were, during the height of the pandemic, administered by telephone and participants were sent the information and materials needed to complete the training tasks and questionnaires by email, with instructions on how to open the files and how and when to perform the tasks and measurements being provided by telephone. The participants’ performance and responses were monitored and documented by the task leader throughout the training period. We resumed testing at the sites, as soon as the national COVID-19 guidelines and protocols of the participating centers permitted us to. Safety measures included the use of plastic screens between test leaders and participants, the use of gloves, and wearing face masks at all times.

### Ethics

The study has been approved by the regional Medical Ethical Committee Arnhem-Nijmegen, the Netherlands (trial number NL67671.091.180). This trial is registered in the Netherlands Trial Register (code: NL7639). The study protocol adheres to CONSORT guidelines (Moher, Schulz, Altman, & Group, 2001). Data will be processed confidentially, with only the principal and coordinating investigators having access to confidential information. Monitoring of the research process will be done regularly, by an independent monitor. Data management is overseen by an independent data management officer.

Adverse events will be defined as any undesirable experiences participants may undergo during the study, irrespective of whether they are (considered to be) related to the CCT or placebo training. All spontaneously reported adverse events and those observed by the investigator will be recorded. Serious, potentially life-threatening events will be reported to the ethics committee within 7 days.

### Interventions

#### aPASAT training

In the adaptive Paced Auditory Serial Addition Task participants are asked to give the sum of the last two numbers of a continuously presented auditory stream of digits (1–9) and type in the corresponding number (2–18) on the tablet screen. Each training session starts with an interstimulus interval (ISI) derived from the participant’s baseline performance, which interval is subsequently adjusted based on his/her current performance to thus modify task difficulty: following four consecutive accurate or inaccurate answers, the ISI is adjusted with +/− 100 ms, after which the training speed is adapted to the participant’s present response speed. Each session consists of 400 trials corresponding to 20 min training time if the average ISI is 3000 ms; training times can thus vary dependent on participants’ response speed.

#### Placebo training

Participants in the control group will be presented with an active placebo training whose duration, frequency and presentation matches the actual CCT. However, participants will only be asked to tap the number on the touch screen that corresponds to the last heard digit, thus not loading working memory. Research has shown this to be a reliable control condition for the active CCT intervention [35].

#### Measures

Table 1 provides an overview of all measurements and their timepoints.

#### Depressive symptoms

##### Ids-SR

The Dutch version of the Inventory of Depressive Symptomatology Self Report [52] will be used to assess the severity of depressive symptoms. It consists of 30 items covering various symptom domains of depression. Apart from the baseline measurement (T0), the questionnaire will be administered within 1 month after CCT completion (T1) to measure the direct effects of CCT, and at three follow-up timepoints (T2, T3, and T4) to evaluate the longer-term effects (up to 12 months). Treatment response will be defined as a 50% IDS-SR symptom reduction between baseline and 12-month follow-up. Depression remission will be defined as an IDS-SR score of ≤14.

#### Rumination and cognitive emotion regulation

##### RRS

The Ruminative Response Scale (RRS) will be used to assess trait rumination [46] at T0 and T1. The Dutch version contains 22 items and demonstrated good internal consistency.

##### BFT

The breathing focus task [28], also administered at T0 and T1 only, serves to measure state rumination. Participants are asked to close their eyes and focus on their breathing for 5 minutes. Throughout, they will hear 12 random sound signals and after each signal they are asked to report whether they are focusing on their breathing. If they are not, they are to rate the nature of the intrusion as positive, negative, or neutral, and describe the thought briefly (e.g., negative - my depression). The number of negative intrusions reflects the severity of rumination.

##### CERQ

To gauge the participants’ cognitive strategies to deal with emotions, the cognitive emotion regulation questionnaire [23] will be used. Its 36 items are divided into nine subscales. In each subscale, the respondent needs to indicate his/her reaction to the threatening or stressful life event that is described. They will complete the CERQ at T0 and T1.

#### Cognitive functioning and emotional inhibition

##### PASAT

A non-adaptive version of the PASAT will be included as a measure of task-specific transfer of the CCT [24]. Comparable to the aPASAT, participants are presented with a continuous auditory stream of numbers of which they have to add the two last-heard digits. The task is presented in three trial blocks with ISIs of 3000, 2000, and 1500 ms, respectively. The task will be completed at T0 and T1.

##### RNG

The random number generation task will serve as an indicator of working memory capacity [48] and prefrontal functioning. Participants are asked to generate numbers sequentially, usually at a rate of one per second, and instructed to avoid ordered sequences (e.g., 1, 2, 3, 4, or 3, 5, 7, 9). Allowable responses are restricted to a specified range (e.g., 1–10). Instructions to the task are follows: “What I would like you to do is attempt to generate a random sequence of numbers between 1 and 10. Imagine, if you will, having these numbers written on pieces of paper placed in a hat. Shake the hat, draw out a piece of paper and read the number. Then put the paper back into the hat, shake it again, and draw another number out, and continue this process”. The task leader notes the responses and response times on an answer sheet. A participant’s working memory capacity is determined by the response times and randomness of the answers given [7], and is related with prefrontal functioning. The RNG is administered at T0 and T1.

##### Stroop task

The task [64] assesses whether participants are able to inhibit attention to interfering stimuli. We will be using both the classical and an emotional Stroop task. In the former task, participants are shown both congruent stimuli (e.g., the word ‘green’ presented in a green color) and incongruent stimuli (e.g., the word ‘green’ presented in a yellow color) on a laptop and instructed to name the color in which the words are presented as fast and as accurately as possible. Three screens are presented: a congruent and an incongruent screen and one with strings of letters printed in different colors (red, yellow, green and blue) that are presented in 12 rows of 4 words providing an index of basic color naming speed. The screens are presented in random order to avoid order effects. The researcher measures the time per screen with a stopwatch. Response times and accuracy serve as measures of inhibition ability.

In the emotional Stroop task words of different valence (i.e., positive, negative, or neutral) are presented in different colors (red, yellow, green, and blue). Words and valances are derived from two databases of Dutch words [10, 41] with one extra neutral word added. The words are presented in a similar manner to the classical Stroop task. The difference in response times and accuracy rates between the dissimilarly valenced cards indicates the participant’s cognitive ability to inhibit task-irrelevant emotional information.

##### Moca

The Montreal Cognitive Assessment will be used to screen for mild cognitive dysfunction at baseline (T0). The test assesses eight domains of cognitive functioning: attention and concentration, executive functions, memory, language, visuoconstructional skills, conceptual thinking, calculations, and orientation [44].

#### Quality of life

##### EQ-5D-5L

The 5-item, 5-level EuroQoL is used to monitor changes in health-related quality of life (in terms of quality-adjusted life years, QALYs) and is completed at T0 and follow-up (T2, T3 and T4) [31, 50].

##### WHODAS 2.0

The World Health Organization disability assessment schedule evaluates the degree of disability based on the ICIDH-2 model of health-related states and functioning. The WHODAS 2.0 determines disability associated with both physical and mental disorders in both general and clinical populations [13]. Recently, an algorithm has been developed to map WHODAS 2.0 scores on disability weights [37]. The WHODAS is administered at all five assessment timepoints.

#### Economic evaluation

##### TiC-P

The Netherlands Institute of Mental Health and Addiction and iMTA Cost Questionnaire for Psychiatry [26] will be administered to assess healthcare utilization, patients’ out-of-pocket costs and productivity losses stemming from absenteeism and lesser productivity when working. The TiC-P is the most frequently used health service receipt questionnaire in the Netherlands. It will be administered at T0 and follow-up (T2, T3, and T4).

### Implementation study

Alongside the RCT, we will conduct a quantitative and qualitative implementation study. The overall goal of the implementation study is to examine acceptability and feasibility of CCT and to identify facilitating and impeding factors in the implementation process. It contains three parts, each part addressing a separate target group:

1. Participants: we will conduct qualitative in-depth interviews in different subsamples of patients (responder/non-responders; drop-out/no drop-out). We aim for 16 to 20 interviews, or until data saturation has been reached. Topics are partly guided by items of the Patient Education Materials Assessment Tool for Audiovisual Materials [55] and will address satisfaction, experienced effects, usability, understandability, support, and clarity of the intervention. Interviews will take place between T2 and T3. In addition, two questions on satisfaction are added to the T1 RCT questionnaire for participants in the experimental group, and the research team will systematically assess the reasons for potential drop-out.
2. Healthcare professionals: the perceptions of the health care professionals (therapists and coordinating professionals in the teams) will be evaluated by conducting a focus group with a subsample of 6 to 8 of them. Topics will include satisfaction, appropriateness, embedding in work routine, acceptability, and facilitating and impeding factors for current and eventual future implementation of CCT in mental healthcare. These topics are based on a review that identified the most important factors that can promote or inhibit implementation of online interventions for mood disorders in routine practice [61]. The focus group will take place at the end of the inclusion phase. Moreover, all healthcare professionals involved will receive an online survey, containing the same topics as in the focus group, as well as the Normalization MeAsure Development (NoMAD) questionnaire: a 20-item self-report instrument based on Normalization Process Theory that measures implementation processes [38, 62].
3. Stakeholders: if the RCT shows (cost-)effective results, we will conduct a focus group interview with all stakeholders in order to identify facilitators and barriers for nationwide implementation. Stakeholders are, again, representatives from the patients and health care professionals, as well as representatives from the Netherlands Ministry of Health, Welfare and Sport, insurance companies, Netherlands Healthcare Authority and the Committee for clinical guidelines in depression treatment. These final results of the implementation study will be summarized in an implementation plan for future rollout.

The implementation study takes place in an iterative process whereby new insights can lead to adjustments in order to ensure optimal inclusion and implementation during the trial.

### Statistical analyses

#### Sample size calculation

A previous power calculation based on MDD studies demonstrated that an effect size of η^2^ = 0.11 or larger should be attainable [35]. This is equivalent to a standardized mean difference of Cohen’s d = 0.70 or larger. In the current study protocol, which is powered based on the expected effect on the primary outcome measure, we prefer to make a more conservative assumption regarding the expected effect size and opt for d = 0.45, corresponding to a medium-size effect, which would be relevant from a clinical point of view. Effects of d = 0.45 (if present) will be statistically significant in a baseline adjusted ANCOVA with one baseline (post-training) and four follow-ups (at 3, 6, and 12 months) at α = 0.05 (2-tailed) and a power of (1-ß) = 0.80. Assuming a correlation of *r* = 0.50 between baseline and first follow-up and again *r* = 0.50 between subsequent follow-ups, we need *n* = 33 per condition to attain a well-powered study.

However, we will also take into account that the data of the participants will be ‘clustered’ within treating clinicians, which introduces a design effect of de = 1.56 when the mean cluster size is m = 10, the coefficient of variation is cv = 0.475, and the intraclass correlation icc = 0.05 [21]. Multiplying the sample size (*n* = 33 per condition) × the design effect de = 1.56 indicates that, at baseline, we need *n* = 52 per condition or *n* = 104 for both arms. We will not compensate for loss to follow-up because we will conduct intention-to-treat analyses (i.e., including all participants as randomized) as per the CONSORT statement – either using (generalized) mixed models or multiple imputation of missing observations.

#### Evaluation of clinical outcomes

All analyses will be conducted in agreement with the intention-to-treat (ITT) principle as per the CONSORT statement [40]. We will repeat the analyses in the per-protocol sample: participants who completed at least six training sessions. We will use two-level generalized mixed models on the longitudinal dataset to analyze the clinical outcomes over all 4 time points with condition, time and the interaction of condition x time as factors and the depended baseline measure as covariate while accounting for the multilevel data structure with participants being ‘nested’ in clinicians and assessments ‘nested’ within participants [27]. We will examine baseline cognitive control, depression severity, and age as possible moderators. We will examine the latter as possible moderating variable, because age was a relevant correlate for the effect of the aPASAT on depressive symptoms [12]. Rumination and working-memory functioning will be evaluated as potential mediators of change in depressive symptoms, for which Hayes’ PROCESS macro (in SPSS) will be used. These evaluations of the clinical outcomes will be carried out in SPSS [30] and Stata [58].

#### Health-economic evaluation

Following the CHEERS statement [29], the health-economic evaluation will be conducted in agreement with the ITT principle on a wide dataset, which will be multiply imputed using chained equations. For the health-economic evaluation, we will take both the healthcare systems perspective and the societal perspective, thus incorporating the intervention costs, all other healthcare costs, the patients’ (and their families’) out-of-pocket costs into account in the healthcare perspective, plus productivity losses (stemming from absenteeism and lesser productivity while at work but feeling ill) for the societal perspective.

In the *cost-effectiveness analyses* incremental costs will be related to (a) IDS-SR treatment response, (b) IDS-SR remission, while in a *cost-utility analysis* the incremental costs will be related to EQ-5D-5L quality adjusted life year (QALY) gained.

To that end, incremental cost-effectiveness ratios (ICERs) will be computed, bootstrapped (2500 times) and the bootstrapped ICERs will be projected on the ICER plane as a scatter of simulated ICERs. When the simulated ICERs largely fall in the NE quadrant of the ICER plane, then the intervention generates better health outcomes than enhanced usual care, albeit for additional costs; in the NW quadrant the intervention costs more than enhanced usual care and generates less health benefits and is therefore said to be ‘dominated’ by usual care; in the SW the intervention costs less but will be associated with fewer health benefits than usual care; finally, in the SE quadrant the intervention dominates care as usual because it is both less costly and is associated with better health outcomes then enhanced care as usual. Thus, the ICER plane facilitates medical decision-making in a probabilistic way.

The bootstrapped ICERs will also be used to graph the ICER acceptability curve depicting how the probability that the intervention should be regarded as cost-effective depends on the willingness-to-pay per (a) treatment responder, (b) remission, (c) QALY gained.

In addition to the cost-effectiveness analysis (CEA) and cost-utility analysis (CUA), we will also conduct a budget-impact analysis (BIA). In the BIA we will assess the budget impact on the public purse of scaling up the intervention’s implementation, e.g. for scenarios where the intervention covers 20, 40, 60 or the full 100% of the intended target group of people seeking treatment for MDD in later life.

Finally, all health-economic evaluations (CEA, CUA and BIA) will be subject to sensitivity analyses with 20% smaller and 20% larger per-participant intervention cost of the add-on cognitive training. This will be done to ascertain the robustness of the main analyses.

## Discussion

The proposed RCT will examine the (cost-)effectiveness of a cognitive control training (CCT) program as an add-on intervention to specialized depression treatment in adults over 60 years coping with late-life depression (LLD). We will be offering the aPASAT as the active intervention since it targets working memory, which has been found to be effective in reducing depressive symptoms, possibly by increasing cognitive control over repetitive negative thought intrusions. Its effectiveness will be compared to TAU coupled with a placebo intervention (sham training). Change in depressive symptoms, potential working mechanisms, and cost-effectiveness will be investigated post-training and three times during a one-year follow-up. Being a pragmatic trial, inclusion restrictions will be limited, to thus to enhance its ecological validity pertinent to depressed populations in real-world clinical settings.

In addition to change in depressive symptoms, we will study change in rumination and cognitive deficits (i.e., reduced working memory performance) as possible working mechanisms of CCT since both cognitive pathways have been shown to be addressed by the aPASAT [8, 19], with rumination having been identified as a mediating factor between executive dysfunction and depression-related behavior in LLD [63]. We will also be making a first exploration into the effect of CCT on cognitive coping and inhibition of irrelevant (emotional) stimuli to examine the role of these underlying factors of LLD. We will also be looking at age, baseline cognitive control and depression severity to see whether these individual factors affect the effectiveness of CCT differentially and to predict which patients might benefit most from the intervention for well-informed personalized prescription or referral. We also hope to establish whether the addition of CCT to TAU for LLD improves health-related quality of life and overall daily functioning, which often remains impaired even after symptom reduction [14].

From a societal perspective, the cost-effectiveness of mental-health innovations is an important factor. We expect online CCT to reduce healthcare consumption in older adults, while, in and of itself, CCT is an inexpensive treatment. It is readily accessible from home and its standardized format allows easy monitoring and guidance, and systematic comparison of outcomes over time.

The results from the flanking implementation study will serve to inform a wider implementation of digital interventions targeting this growing population within and outside mental-health care, potentially lowering the need for more expensive face-to-face health services. Since the target demographic will become increasingly familiar with digital technologies, computerized interventions will become increasingly relevant, a trend which has been amplified by the COVID-9 pandemic and its ensuing lock-downs. In the implementation study, we will schedule qualitative interviews and focus group meetings to evaluate the appropriateness, acceptability and referral of online CCT. The goal of the implementation study is sustainable later scale up of the CCT intervention. Previous results on the acceptability of digital CCT formats in this population were promising [6], but findings were often obtained in smaller pilot studies. The data we will be collecting will not only support an adequate implementation of the intervention under study but also offer insight into the acceptability and practical implications of digital interventions in general.

Despite the strengths of the trial, there are some limitations. First, there is no control group that receives only treatment as usual to monitor the natural course of depressive symptoms. Both groups follow a form of CCT – active or placebo. Therefore, the results of this trial will not be able to compare CCT treatment to TAU alone. Second, most patient will likely be treated within specialized mental healthcare centers, which limits the generalizability of the results.

In conclusion, new treatment strategies for late-life depression are urgently needed and we hope that, if proven effective, feasible and cost-effective, the intervention will be added to (inter)national guidelines as a recommended add-on to current depression treatment for older adults.

## Data Availability

Not applicable.

## Abbreviations

LLD: Late-life depression
CCT: Cognitive control training
TAU: Treatment as usual
RCT: Randomized controlled trial
NESDO: The Netherlands Study of Depression in Older Persons
CCN: Cognitive control network
dACC: Dorsal anterior cingulate cortex
DLPFC: Dorsolateral prefrontal cortex
(a)PASAT: (adaptive) Paced Auditory Serial Addition Task
DSM-IV: Diagnostic and Statistical Manual of Mental Disorders
M.I.N.I.: Mini International Neuropsychiatric Interview
SCID-I: Structured Clinical Interview for DSM-5
MDD: Major Depressive Disorder
MMSE: Mini-Mental State Examination
IDS-SR: Inventory of Depressive Symptomatology – Self Report
TiC-P: Trimbos Institute and iMTA Cost questionnaire for Psychiatry
COVID-19: Coronavirus disease 2019
RRS: Ruminative Response Scale
BFT: Breathing Focus Task
CERQ: Cognitive Emotion Regulation Questionnaire
RNG: Random Number Generation task
MOCA: Montreal Cognitive Assessment
EQ-5D-5L: EuroQol five-dimensional five-level questionnaire
QALY: Quality-adjusted life-years
WHODAS 2.0: The World Health Organization disability assessment schedule
NoMAD: Normalization MeAsure Development
CONSORT: Consolidated Standards of Reporting Trials
CHEERS: Consolidated Health Economic Evaluation Reporting Standards
ITT: Intention to treat
ICER: Incremental cost-effectiveness ratio
CEA: Cost-effectiveness analysis
CUA: Cost-utility analysis
BIA: Budget-impact analysis
